# Mitigating bias in the analysis and inferences from using longitudinal EHR data in disease outcomes research

**DOI:** 10.1186/s12874-026-02881-6

**Published:** 2026-06-11

**Authors:** Cassandra Hennessy, Alison Z. Swartz, Frank E. Harrell, Heidi J. Silver

**Affiliations:** 1 Department of Biostatistics, Vanderbilt University Medical Center, Nashville, TN USA; 2 Department of Medicine, Vanderbilt University Medical Center, Medical Arts Building Suite 214, 1211 21st Avenue South, Nashville, TN 37232 USA; 3 Department of Veterans Affairs, Tennessee Valley Healthcare System, 1310 24th Avenue South, Nashville, TN 37212 USA

**Keywords:** Weight, BMI, Cardiovascular, Weight cycling, EHR, Outcomes

## Abstract

**Background:**

The electronic health record (EHR) provides an opportunity for extracting a wealth of up-to-date real-world longitudinal data. Although the main limitations of using EHR data have been well-recognized and well-described, under-recognized factors may threaten the reliability of inferences made regarding the impact of EHR variables on chronic disease outcomes. A problem not well-recognized is the impact of routinely acquired variables that are documented with every patient encounter regardless of the reason for the encounter such as vital signs, height, and body weight.

**Methods:**

We utilized a landmark approach to identify occurrence of 10 cardiovascular-related disease outcomes after a 5-year observation period during which all body weights recorded in the EHR were used in multivariate cox regression modeling to identify the strongest of 9 weight-based predictor variables for each of the 10 disease outcomes.

**Results:**

We found that the number of recorded weights, as an independent variable, was the strongest predictor for all 10 cardiovascular-related disease outcomes when compared to all other weight-based variables (lowest weight, highest weight, average weight, last weight, absolute weight change, maximum weight change, weight fluctuation, and weight cycling) as well as BMI. The findings demonstrate the importance of recognizing and accounting for the number of times a more frequently measured clinical variable, such as body weight, is recorded as it is critical to determine the true impact of other similar variables on disease outcomes when conducting longitudinal analysis of EHR data.

**Supplementary Information:**

The online version contains supplementary material available at 10.1186/s12874-026-02881-6.

## Introduction

The electronic health record (EHR) provides a rich source of up-to-date real-world longitudinal data that can be readily extracted for investigating the prevalence, treatments, and outcomes of chronic diseases. The usage of EHR data in clinical and translational research is particularly important because assessing the impact of patients’ trajectories, identifying trends, and capturing patterns is challenging in situations where a long-term prospective protocol for routine data collection is not feasible. In addition, the EHR provides access to a large dataset incorporating a wide variety of variables (e.g., demographics, vital signs, anthropometrics, medications, medical and surgical history, laboratory and test results, procedure reports, diagnoses, and treatments) that can inform guidance and policies. Moreover, data can be anonymized to protect patient privacy and security. Further, EHR data extraction can be performed straightforwardly and immediately, and therefore, limit research costs.

Despite the multiple benefits of using EHR data for research, several challenges exist regarding the quality and interpretation of EHR data that may threaten the reliability of inferences made on the impact of EHR variables on health conditions and disease outcomes. The main limitations of using EHR data have been well-recognized and well-described, and generally stem from the fact that the EHR is designed to be a documentation and billing tool, not for conducting longitudinal research. [1–5] For one, the EHR contains not only structured but unstructured data such as free-text notes and summaries that can be challenging to analyze. Secondly, there may be missing data and misclassifications as well as data entry, recall, and documentation errors. Exacerbating these issues, the billing codes used may vary between health care providers, leading to bias in which disorders are reported in EHR studies. [6] Beyond missingness and random errors, socioeconomic and health behavior data such as substance use, dietary intake, and physical activity may be inconsistently assessed or entirely undocumented. Additionally, the patient data available in the EHR may not be entirely representative of the research population targeted.

A problem not well-recognized in the published evidence base is the impact of routinely acquired variables that are documented with every patient encounter regardless of the reason for the encounter such as vital signs, height, and body weight. While these data are informative, the frequency they are being recorded not only reflects that a patient is having more health care encounters, and thus more health-related concerns, it also adds an additional challenge to the interpretation and inferences made from longitudinal analysis that are not adequately resolved by employing typical repeated measures statistical methodology.

Here we aim to explore the nature and extent of this particular type of bias from confounding by applying advanced statistical modeling techniques to a longitudinal dataset extracted from the Vanderbilt University Medical Center (VUMC) EHR which stores patient data from 7 hospitals and > 180 outpatient clinics throughout Tennessee and nearby states. To do so, we targeted body weight as a proxy for any other routinely recorded biomarker as it is a robust and independent risk factor that is utilized in screening for several chronic diseases, especially cardiometabolic diseases, as well as other comorbidities and mortality. Thus, we aimed to determine whether the number of weights recorded in the VUMC EHR over a 5-year observation period is the dominating predictor variable for cardiovascular-related disease states when compared to real-time body weight measures as predictive factors. We propose that measurement frequency is an under-recognized but critical source of confounding in EHR research. Incorporating a more rigorous analysis process to account for the number of times a variable such as weight is recorded is a complex but necessary task as not accounting for the potential impact from the frequency of a recorded independent predictor variable such as body weight (or other vital sign measure) may produce misleading findings that can affect clinical decision making for patient care or public health policies.

## Methods

### Patient Data

Demographic (age, sex, self-reported race and ethnicity), anthropometric (height, weight, BMI), smoking status, and medication data were extracted from the synthetic derivative of the VUMC EHR for all adult patients treated during the years 1997 through 2020 (Supplemental Fig. 1). Patients were excluded if they were under 18 years old, had documentation of any malignancy other than non-melanoma skin cancer, had a history of bariatric surgery, were pregnant, had no documented height, had an implausible BMI of < 15 or > 80 kg/m^2^, or had less than 5 years of successive weights documented with each weight no more than 18 months from the prior weight. These exclusions led to a final cohort of 83,261 VUMC patients.

### Predictors and outcomes

We used a landmark approach to identify the occurrence of any of 10 cardiovascular-related disease outcomes after a 5-year observation period during which all recorded weights were integrated into a multi-stage data cleaning algorithm that also incorporated linear interpolation methods to create a standardized grid of the weights every 60 days across the 5-year observation period. A combination of ICD-9/10 codes, CPT codes, and natural language processing were used to identify the presence/absence of the 10 cardiovascular-related disease outcomes: peripheral artery disease (PAD), coronary artery disease (CAD), cardiac catheterization (CC), heart failure (HF), myocardial infarction (MI), obstructive sleep apnea (OSA), hypertension (HTN), hyperlipidemia (HLD), metabolic-associated steatotic liver disease (MASLD), and type 2 diabetes (T2D), as previously described. [7] Patients with the outcome being modeled prior to the landmark demarcation point (end of year 5) were censored from analysis of that outcome. The landmark period was completed at the end of the year 2020 or when a patient had no more entries in the VUMC EHR (Supplemental Fig. 2). Full details of the data acquisition, cleaning, and standardization methods have been reported in our prior publications. [7, 8].

In addition to the number of weights recorded, we investigated the predictive value of 8 other body weight indicators available from data collected in the EHR during the 5-year observation period: lowest weight recorded, highest weight recorded, average weight recorded, last weight recorded, absolute weight change, maximum weight change, weight fluctuation, and weight cycling in order to determine the most robust weight-based predictor for each disease outcome. Weight fluctuation was defined by average successive weight variability (ASV). [9] Weight cycling was defined as having one or more periods of ≥ 5% weight loss and regain within the 5-year observation period. [10].

### Statistical analysis

Analyses were performed using R version 4.3.2. (R Foundation for Statistical Computing, Vienna, Austria). A multi-stage iterative approach was taken in performing multivariate Cox regression modeling for each of the 10 cardiovascular-related disease outcomes. Chi-square statistics corrected for the number of degrees of freedom were produced to evaluate the contributions of predictor variables to the regression models. All four models were adjusted for the potentially confounding factors of age at baseline, sex, self-reported race and ethnicity, height, baseline BMI, smoking status (never/ever/unknown), medications for dyslipidemia, hypertension and hyperglycemia, and the number of weights recorded during the 5-year observation period. First, to obtain a global ranking of importance for each of the 10 cardiovascular-related disease outcomes independently, the 8 weight-based predictor variables were ranked from 1 to 8 with the rank of 1 indicating the strongest weight-based predictor for an outcome based on its chi-square minus degrees of freedom value. A mean rank score was then calculated for each weight-based variable across the 10 outcomes to create an overall global ranking which determined the sequential order in which the 8 weight-based variables would be added to regression models (Supplemental Table). The first cox regression model was a partially adjusted model, adjusted only for the potentially confounding factors but unadjusted for the 8 weight-based variables, to determine the strength of the number of weights recorded as an independent predictor for each disease outcome as compared to each weight-based variable. The second model was adjusted for both the potentially confounding factors and each weight-based predictor variable to determine the strength of each weight-based predictor in comparison to the number of recorded weights as an independent variable. For the third model, weight-based predictors were added one at a time to determine the contribution of the chi-square value minus degrees of freedom for each weight-based variable. Chunk tests were employed to determine the cumulative chi-square value for all the weight-based predictors as each weight-based variable was added sequentially when accounting for the confounding factors and the number of recorded weights. The fourth (final) model was performed to assess how much each weight-based variable contributes to the overall model based on its adjusted chi-square minus degrees of freedom value while accounting for both the potentially confounding factors and the weight-based variables. The dotchart() function in R was used to create Cleveland dot plots comparing the chi-square minus degrees of freedom and *p* values among predictor variables for each cardiovascular-related disease outcome. In addition, bootstrap estimates of calibration accuracy for 5-year estimates for each of the full Cox models were conducted (Supplemental Fig. 3). These show the bias-corrected estimates of predicted survival probability as a single time point versus the corresponding Kaplan-Meier estimate. As observed from the overfitting-corrected calibration curve in comparison to the apparent calibration, our large effective sample size (essentially the number of events) led to minimal overfitting in our models which supports confidence in our findings.

## Results

As previously reported, the 83,261 unique patients had a median of 14 (IQR: 10, 21) weights recorded during the 5-year observation period, with those in the highest quartile of ASV having a median of 23.0 (IQR: 16, 34) recorded weights. At the beginning of their 5-year observation period, the median BMI was 27.6 kg/m^2^ (IQR: 24.1, 32.1) with 78% of males and 63% of females meeting BMI criteria for having overweight (25.0–29.9 kg/m^2^) or obesity (≥ 30 kg/m^2^). The median length of the landmark follow-up period was 5.2 (IQR: 3.3, 8.0) years. As presented in Table 1, the median body weight during the landmark period was 80.98 kg (IQR: 67.61, 95.96) with a highest recorded weight of 86.18 kg (IQR: 72.29, 102.10) and a lowest recorded weight of 74.90 (IQR: 62.60, 89.36). More than half (54.4%) of patients had at least one bout of weight cycling and the median ASV was 0.69 (IQR: 0.45, 1.05). The incidence of patients being diagnosed during the follow-up period with a cardiovascular-related disease was 10.2% for hyperlipidemia, 8.8% for hypertension, 6.5% for obstructive sleep apnea, and 5.2% for type 2 diabetes (Table 2).

**Table 1:** Median [IQR] of weight-related predictor variables

Weight Cycling (%)	45,294 (54.4%)
Weight Fluctuation (ASV)	0.69 [0.45, 1.05]
Last Recorded Weight (kg)	81.42 [67.95, 96.62]
Absolute Weight Change (kg)	0.91 [-3.17, 5.04]
Highest Recorded Weight (kg)	86.18 [72.29, 102.10]
Lowest Recorded Weight (kg)	74.90 [62.60, 89.36]
Average Weight (kg)	80.98 [67.61, 95.96]
Maximum Weight Change (kg)	4.25 [-8.67, 10.36]
Number of Weights (no.)	14.00 [10.00, 21.00]

**Table 2:** Cumulative incidence of 10 cardiovascular-related disease outcomes during landmark follow-up period

Total *N*	83,261
Peripheral Artery Disease (PAD)	2151 (2.6%)
Coronary Artery Disease (CAD)	3654 (4.4%)
Cardiac Catheterization (CC)	3507 (4.2%)
Heart Failure (HF)	2580 (3.1%)
Myocardial Infarct (MI)	3147 (3.8%)
Hypertension (HTN)	7336 (8.8%)
Hyperlipidemia (HLD)	8528 (10.2%)
Type 2 Diabetes (T2D)	4365 (5.2%)
Metabolic-Associated Steatotic Liver Disease (MASLD)	2447 (2.9%)
Obstructive Sleep Apnea (OSA)	5417 (6.5%)

**Fig. 1:**
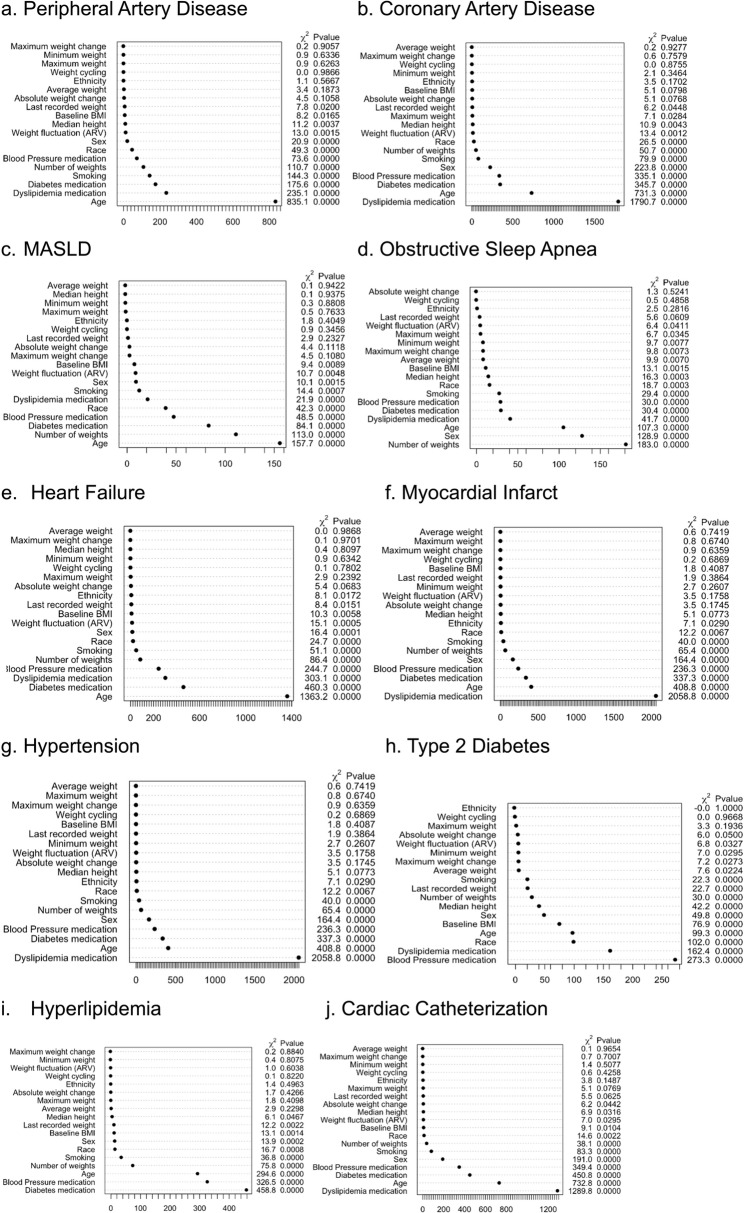
**(a-j).** Cleveland dot plots comparing chi-square minus degrees of freedom values from Cox regression modeling of independent predictors for 10 cardiovascular-related disease outcomes

Results from the partially adjusted models (Table 3 Model 1) showed the number of weights recorded in the EHR was the strongest predictor for 8 of the 10 cardiovascular-related disease outcomes (PAD, CAD, CC, HF, MI, HTN, HLD, MASLD). Further supporting this finding, the number of recorded weights was the strongest predictor for all 10 cardiovascular-related disease outcomes in the fully adjusted models (Table 3 Model 2). While adjusting for the potentially confounding demographic and clinical factors decreased the strength of the predictive effect of the number of recorded weights as an independent variable, the only factors that consistently had higher chi-square values predicting the incidence of a cardiovascular-related disease than the number of recorded weights were age and medications for blood pressure, dyslipidemia or hyperglycemia control (Fig. 1).

**Table 3:**
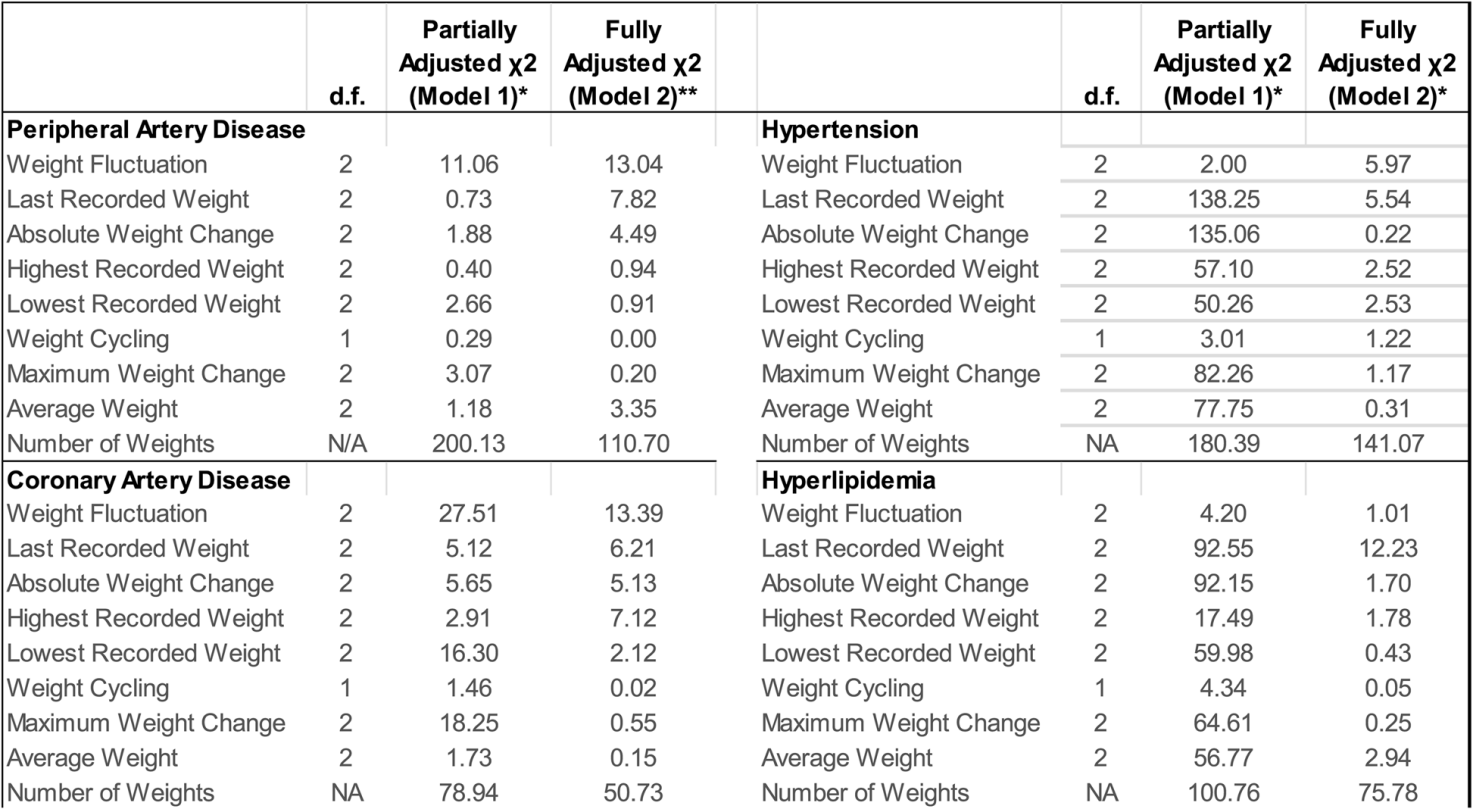

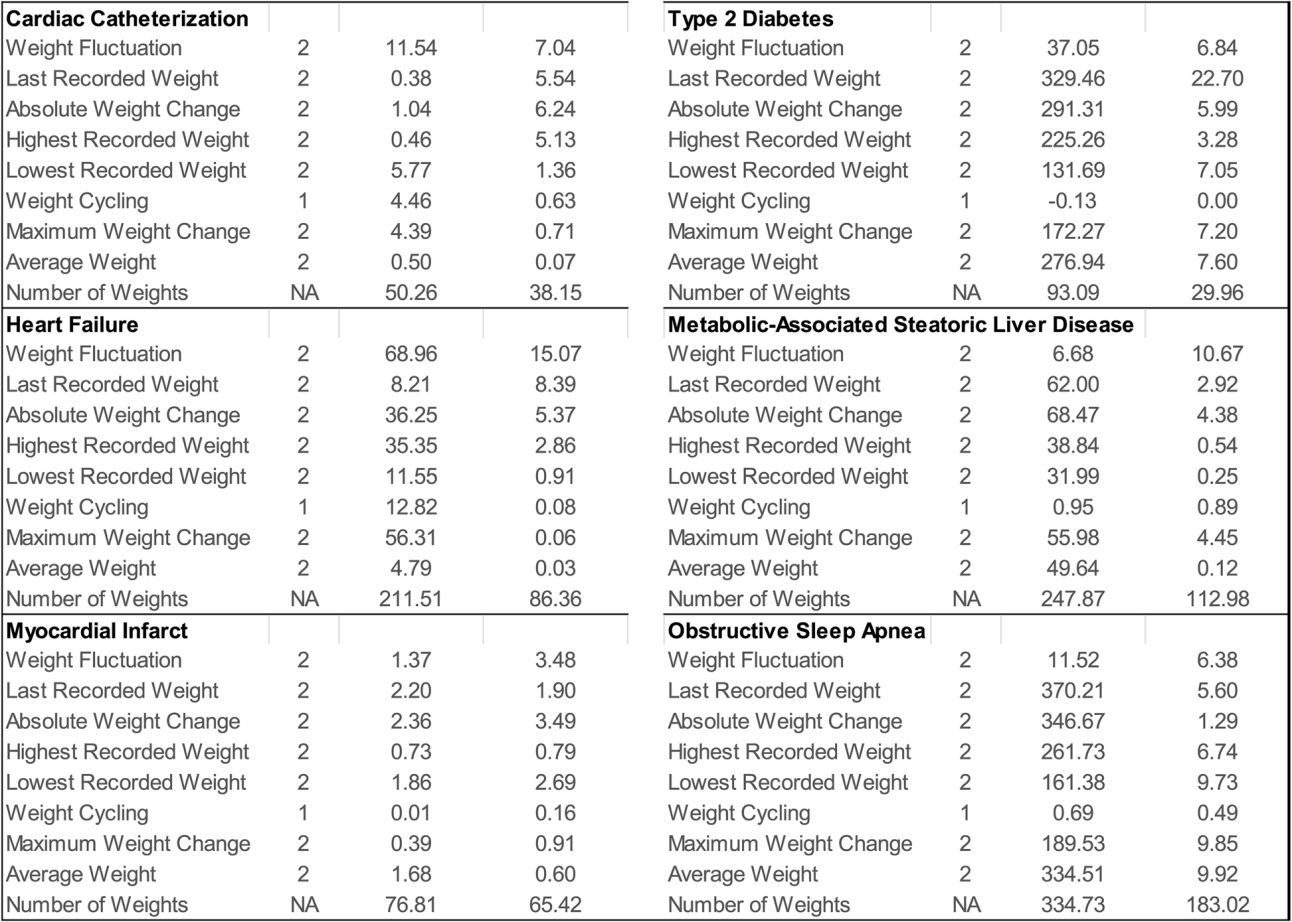
Results from partially and fully adjusted cox regression modelling by cardiovascular disease-related condition

Model 1 adjusted for age, sex, race, ethnicity, baseline BMI, height, medications, smoking status, and number of recorded weights. Model 2 adjusted for age, sex, race, ethnicity, baseline BMI, height, medications, smoking status, and number of recorded weights and each weight predictor is adjusted for all other weight-related variables

Weight fluctuation (an indicator of weight variability or instability) was the second strongest predictor in the partially and fully adjusted models for PAD, CAD, CC, HF, MI, HTN, and MASLD whereas the last recorded weight was the second most robust predictor for HLD and T2D. In the third regression model (Table 4 Model 3), wherein weight-related variables were added sequentially based on global ranking score, the highest recorded weight contributed most to the predictive value of the model after weight fluctuation for PAD, CAD, and CC. In contrast, absolute weight change contributed most after weight fluctuation for HF whereas it was lowest recorded weight for MI and last recorded weight for HTN, HLD, T2D, MASLD and OSA. The final model (Table 4, Model 4) reveals the cumulative chi-square value for each cardiovascular-related disease state as each weight-related variable is added sequentially.

**Table 4:**
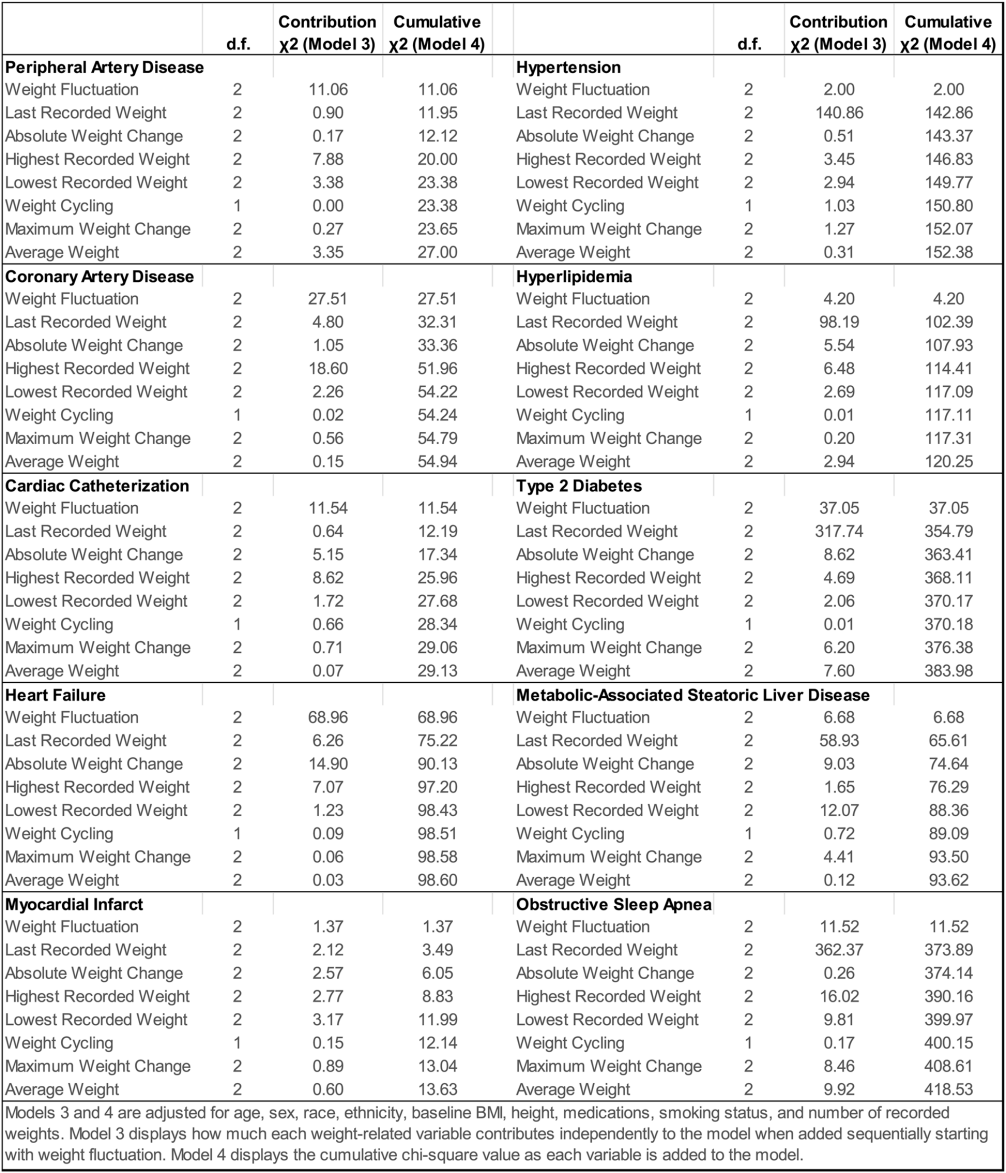
Results from cox regression modelling to determine contribution to chi-square value of weight-related variables of each cardiovascular disease-related condition

Models 3 and 4 are adjusted for age, sex, race, ethnicity, baseline BMI, height, medications, smoking status, and number of recorded weights. Model 3 displays how much each weight-related variable contributes independently to the model when added sequentially starting with weight fluctuation. Model 4 displays the cumulative chi-square value as each variable is added to the model

## Discussion

Here we utilize the established relationship between body weight and cardiovascular-related disease outcomes [11–13] as an instrument for demonstrating that the number of times a frequently measured clinical variable such as body weight is recorded in the EHR dominates as a predictive factor in the longitudinal analysis of chronic disease outcomes. While the other weight-related variables studied (most consistently weight fluctuation) showed significant predictive probability based on chi-square values for incidence of several cardiovascular-related disease outcomes, none had the strength of the effect that was observed for the number of weights recorded. It is noteworthy that the weight-related variables studied spanned the gamut of potential weight-based conditions: lowest weight, average weight, highest weight, weight fluctuation, weight cycling, last recorded weight, as well as maximum and absolute weight change. Moreover, the number of weights recorded was a stronger predictor for all 10 cardiovascular-related disease outcomes than the cumulative effect of all the other weight-related variables combined. The finding that the number of weights recorded was the strongest weight-related predictor was further confirmed when we consider the chi-square values for baseline BMI, a weight-related variable frequently measured and studied in assessing cardiovascular and chronic disease outcomes, and this was constant for all 10 cardiovascular-related disease outcomes.

Similarly to the number of recorded weights, body weight fluctuation was also a stronger predictor than BMI for the incidence of PAD, CAD, CC, MI, HF, HTN and MASLD. Clinically meaningful fluctuations in body weight, characterized by bouts of weight gain followed by weight loss or weight loss followed by weight regain, have been associated with significantly increased risk for coronary heart disease morbidity and mortality, [14–16] other cardiovascular-related events, [9] and compound CVD outcomes. [13] Further, findings from meta-analysis suggests weight fluctuation is associated with a 40–50% increased risk for all-cause mortality. [17], [18] However, the impact of weight fluctuation on health remains a topic of scientific debate as two recent systematic reviews of the literature assessing the relationship between weight fluctuation and unfavorable body composition showed either no association or inconclusive results. [19, 20] While lack of a standard definition, differences in populations studies, and varied measurement methods may help explain the discrepancies in the published evidence, the conflicting findings also suggest that more statistical rigor is needed including adjustment for the number of times weight is measured when performing longitudinal analysis of EHR data as it may be the confounding factor that drives the cardiometabolic risk that has been associated with weight fluctuation.

Further supporting our findings regarding the impact of a frequently measured and unplanned variable, evidence from autism spectrum disorder research shows that differences in the frequency of contact with the health care system promotes a type of information bias that influences the impact on statistical associations with comorbidities of the disorder indicating that some of the associations reported may be spurious. [21] Other scenarios and simulations conducted to study informative presence bias also show that variability within the biomarker being studied promotes potential for over or under-estimation of effect on outcomes. [22] In the current project, we aimed to extend prior work by exploring a variable commonly measured and recorded in EHR data, regardless of the reason for a health care encounter, and one that is strongly associated with several chronic disease states including cardiovascular diseases which remain the number one leading cause of death.

In contrast to including the number of times a clinical variable is recorded in the EHR, most epidemiological studies investigating incidence or risk for cardiovascular and other chronic disease outcomes using routinely measured clinical variables perform analyses either using a single time point of the measured variable or they do not account for the frequency that the variable is measured when using all available repeated measures over a period of time. [23] Omitting a confounding variable like the number of recorded weights, termed unmeasured confounding, would bias the estimate of the effect of the independent predictor variable studied, such as weight fluctuation or other weight-related independent variables. The degree to which the number of recorded weights changes the relationship between other weight-related variables and the cardiovascular-related disease outcomes can be observed by statistical methods to adjust for the number of weights recorded. Unmeasured confounding typically results in bias in the estimation of the effect of an exposure that increases as the amount of unmeasured confounding increases, as revealed in a simulation study using logistic regression to determine the amount of bias that can occur due to unmeasured confounding. [24] However, there are also cases where the effect estimate of an exposure is reduced by unmeasured confounding. Either way, unmeasured confounding distorts the effect of an independent variable or exposure on an outcome.

It is plausible that the type of bias imparted by unmeasured confounding may not be fully corrected using the “gold standard” linear mixed-effects models or generalized estimating equations or survival time-to-event analysis. We suggest here that when conducting longitudinal analysis using EHR data a more advanced methodology may be necessary. For example, joint modeling that combines mixed effects modeling with survival analysis which could handle interdependent data might be a better approach. Other methods for correcting this type of bias have also been proposed including quantitative bias analysis as well as machine learning approaches. We recognize that external validity of the findings presented here may be limited as this study is based on a single healthcare network (VUMC). There remains need for replication of these findings across other healthcare systems, especially those with dissimilar patient populations. Further, there may be participants included in analyses that had subclinical disease or other risk factors for a specific disease outcome but were included in analyses as they did not meet the strict criteria used to identify having the outcome prior to the landmark period.

## Conclusions

We demonstrate here the importance of recognizing and accounting for the number of times a frequently measured clinical variable such as body weight is recorded in longitudinal analysis of data extracted from the EHR. We posit doing so is critical to determine the true impact of other similar variables, in the present case this includes other commonly measured weight-related variables such as weight fluctuation, weight cycling, highest weight recorded, last weight recorded, or BMI on chronic disease outcomes. While it may be supposed that the number of times a variable such as weight is recorded in the EHR simply represents the unhealthiness of a patient, it may also be that the number of visits with recorded weights is a better reflection of the likelihood that patients who are seen more frequently are more likely to receive diagnoses and interventions for their health conditions which would artificially make it appear that they are sicker.

## Supplementary Information

Supplementary file 1.

## Data Availability

Data described in the manuscript and analytic code may be made available upon reasonable request and completion of a data-use agreement.
