# Supplementary material for: Mitigating bias in the analysis and inferences from using longitudinal EHR data in disease outcomes research
Source: BMC Med Res Methodol. 2026 Jun 11;26:181. doi: 10.1186/s12874-026-02881-6 (PMC13480091; doi:10.1186/s12874-026-02881-6)
Supplement: Supplementary file 1 [file 12874_2026_2881_MOESM1_ESM.docx]

**Supplemental Table 1.** Descriptive Characteristics of Study Sample

|  | **Males**  **(N = 33,060)** | **Females**  **(N = 50,201)** |
| --- | --- | --- |
| **Age (years)** | 51.25 ± 15.04 | 49.00 ± 15.78 |
| **Height (cm)** | 178.79 ± 7.40 | 163.59 ± 6.77 |
| **Weight (kg)** | 93.58 ± 20.05 | 76.06 ± 19.71 |
| **BMI (kg/m^2^)** | 29.21 ± 5.70 | 28.40 ± 7.09 |
| **Race (self-reported as White)** | 89% | 85% |
| **Smoking Status (ever Smoker)** | 38% | 25% |

**Supplemental Table 2**. Global Ranking of Weight-Related Variables by Chi-Square Value for Each Cardiovascular-Related Disease Outcome to Determine the Order that Weight-Related Variables Are Added Sequentially in Cox Regression Modeling.

|  | **PAD** | **CAD** | **CC** | **HF** | **MI** | **MASLD** | **OSA** | **HTN** | **T2D** | **HLD** | **Mean Rank Score** |
| --- | --- | --- | --- | --- | --- | --- | --- | --- | --- | --- | --- |
| **Weight Fluctuation (ASV)** | 1 | 1 | 1 | 1 | 2 | 1 | 5 | 1 | 5 | 6 | 2.4 |
| **Last Recorded Weight** | 2 | 3 | 3 | 2 | 4 | 4 | 6 | 2 | 1 | 1 | 2.8 |
| **Absolute Weight Change** | 3 | 4 | 2 | 3 | 1 | 3 | 8 | 8 | 6 | 4 | 4.2 |
| **Highest Recorded Weight** | 6 | 2 | 4 | 4 | 7 | 6 | 4 | 4 | 7 | 3 | 4.7 |
| **Lowest Recorded Weight** | 7 | 5 | 6 | 6 | 3 | 7 | 3 | 3 | 4 | 7 | 5.1 |
| **Weight Cycling** | 5 | 6 | 5 | 5 | 5 | 5 | 7 | 5 | 8 | 5 | 5.3 |
| **Maximum Weight Change** | 8 | 7 | 7 | 7 | 6 | 2 | 2 | 6 | 3 | 8 | 5.8 |
| **Average Weight** | 4 | 8 | 8 | 8 | 8 | 8 | 1 | 7 | 2 | 2 | 6.0 |

**Supplemental Table 3.** Occurrence of cardiovascular-related disease outcomes in total sample during the observation period following the landmark date.

|  | N = 83,261 |
| --- | --- |
| Peripheral arterial disease | 2151 (2.6%) |
| Coronary artery disease | 3654 (4.4%) |
| Heart failure | 2580 (3.1%) |
| Myocardial infarction | 3147 (3.8%) |
| Cardiac catheterization | 3507 (4.2%) |
| Hyperlipidemia | 8528 (10.2%) |
| Fatty liver disease | 2447 (2.9%) |
| Hypertension | 7336 (8.8%) |
| Diabetes | 4365 (5.2%) |
| Sleep apnea | 5417 (6.5%) |
| *Note: 66.6% of patients had none of the outcomes, 22.1% had 1 outcome, 6.8% had 2 outcomes, and 2.7% had 3 outcomes. | |

**Supplemental Figure 1. Flow Chart of Study Eligibility.**

**Supplemental Figure 2. Study Timeline**

**Supplemental Figure 3. Calibration accuracy for 5-year estimates for each of the full Cox regression models.**

**Supplemental Figure 3. Continued**
